# Psychological Femininity and Masculinity and Motivation in Team Sports

**DOI:** 10.3390/ijerph192315767

**Published:** 2022-11-26

**Authors:** Łukasz Bojkowski

**Affiliations:** Department of Psychology, Poznan University of Physical Education, Królowej Jadwigi 27/39, 61-871 Poznań, Poland; bojkowski@awf.poznan.pl

**Keywords:** psychology, femininity, masculinity, motivation, team sports

## Abstract

It is hypothesized that levels of femininity and masculinity may be relevant to specific types of engagement in action. For this reason, the aim of this study was to search for relationships between psychological dimensions of femininity and masculinity and different forms of motivation, as well as their specific parts, among women and male athletes practicing team sports games. We researched 49 women aged 19 to 32 years representing sports such as football, handball, hockey, volleyball, and basketball and 56 men aged 18 to 31 years practicing football, hockey, volleyball, basketball, and handball. The respondents completed the Inventory to Assess Psychological Gender (IPP) and the Polish adaptation of the Sport Motivation Scale (SMS). It was determined that the psychological dimension of femininity was (in the male group) positively related to the dimension of amotivation, i.e., the lack of perception of a relationship between one’s action and the outcome. In turn, the psychological dimension of masculinity was positively related to the motivation to know, motivation to accomplish, and motivation to experience stimulation, as well as the overall level of intrinsic motivation and the overall dimension of extrinsic motivation. Furthermore, the masculinity dimension is, in male athletes, related to the level of the introjection motive, i.e., the process of integrating accepted patterns.

## 1. Introduction

The discussion on sexuality is most often reduced to two terms: sex and gender. The first corresponds to the position of biological essentialism [1], referring to physiological properties, resulting from hormones, chromosomes, or anatomical features of the genital organs. The second term refers to social constructivism, which defines sociocultural gender, resulting from learning the attitudes and behaviors stereotypically ascribed to each gender in a given culture [2,3]. One of the leading concepts derived from social constructivism [1] is gender schema theory [4,5,6], which states that each individual can be ‘characterized’ by two independent [7,8,9,10], not mutually exclusive dimensions—femininity and masculinity [2,4]. According to Bem [6], both cognitive constructs can be variable over time and are revealed by the dominance of one of them [1]. According to contemporary researchers, both constructs are used as labels for attributes attributed to one gender or the other [11].

Depending on biological conditions and cultural patterns, the gender schema may occupy a more peripheral or central position in the structure of the self. Thus, an individual’s characteristic level of psychological femininity and masculinity, shaped and sustained by cultural norms [12], may be important for the quality of their functioning, commitment, and preferences for a course of action (prototyping of experiences) [13]. This may be indicated by research identifying that individuals who possess high levels of both femininity and masculinity (androgynous) have a broader behavioral repertoire, show greater performance in situations requiring independence [14], and function best in cognitive or social terms [15,16]. In contrast, individuals who are characterized by poorly formed traits of psychological femininity and masculinity (sexually indeterminate) are characterized by low self-esteem, lower social openness, and lower sensitivity than representatives of other types [17]. These reports are consistent with the perceived traits and tasks supposedly associated with gender, where the culturally considered schema of femininity tends to include attributes such as a willingness to soothe hurtful feelings, nonuse of coarse language, limited expression of anger and aggression, emotionality, focus on normative standards of beauty, and a shift in priorities to interpersonal relationships [18,19]. In contrast, traditional conceptions of masculinity include analytical, competitive, and defensive views, individualism, a willingness to take a stand on issues, acting as a leader, aggressiveness, self-confidence, resourcefulness, or task orientation [12,20].

On the basis of theoretical reports, it can be assumed that the level of femininity and masculinity—in our case in athletes training team sports games—may be relevant to the type of task engagement [21,22]. It can also influence the type of motivation manifested, related to innate developmental requirements, as alluded to by self-determination theory (SDT) [23,24]. Accordingly, individuals undertake specific activities primarily to satisfy basic psychological needs (autonomy, competence, and attachment). This means that, in the process of self-regulation, individuals are ready to assimilate the rules and social rules of a given culture, which may become apparent in sports competition. In SDT, three forms encompassing the motivation process are detailed [23,24,25]:

1. Intrinsic motivation manifests itself when the cause of the subject’s action is their internal drive, which is not based on external coercion [23,24,25], gives satisfaction, and forms self-confidence and a sense of agency. It is based on the full internalization of rules related to the situation and the environment [26]. Among the specific parts of intrinsic motivation, we distinguish three motives:
  A. Motivation to know—referring to factors such as curiosity, exploration, the desire to learn, and the inner need for knowledge.
  B. Motivation to accomplish—mastery, efficiency, and task orientation.
  C. Motivation to experience stimulation—engaging in an activity with the aim of feeling excited or experiencing exciting sensations.
2. Extrinsic motivation is where the athlete engages in activity on the basis of external factors (rewards and punishments). In extrinsic motivation, partial internalization may occur when external rules have been adopted by the individual but do not constitute their motivational system [23,25]. Among the specific parts of extrinsic motivation, we distinguish three motives:
  A. Identification—valuing one’s action as important.
  B. Introjection—integrating accepted norms, patterns, and rules into the subject’s personality.
  C. External regulation—defining actions subject to the control of external sources.
3. Amotivation is a form of motivation in which a person does not perceive a relationship between their action and the outcome [24].

Analyses justifying differences in the types of motivation of individuals of different genders and different levels of femininity and masculinity can be found in contemporary research [27]. Some of them indicate that, in combat settings, males, compared to women, are more likely to obtain a goal through physical aggression [28,29,30]. Such an effect is often explained by an evolutionary perspective [31,32,33], where men tend to engage in direct competition more often than women [34,35], as well as risk taking in sporting activities [36,37,38], and they are more likely to exhibit ego orientation [39,40,41], which may be related to elevated scales in the psychological masculinity dimension, consistent with characteristics of this dimension. Women, on the other hand, engage more often than men due to their desire to perform the task correctly [42,43,44]. According to researchers, gender differences and cultural gender stereotyping are particularly noticeable in combat sports and team sports games [45,46], which can be equated with warfare and hunting [47].

Summing up, it is emphasized that the level of femininity and masculinity characteristic of a subject may affect the type of their involvement in selected activities [48,49]. This is due to the association of psychological masculinity with a greater tendency—than in the case of psychological femininity—toward aggressive behavior [28,29,30], direct competition [34,35], risk [35], or focus on improving skills, in order to present them to others (competition) [39]. For this reason, we assume that the dimension of masculinity may be correlated with the dimensions of internal motivation (due to the psychosocial profile of team sports games, requiring improvement under long-term training conditions). For this reason, the main aim of the study was to search for relationships of these dimensions with various forms of motivation and their specific parts, among athletes of both sexes training team sports. The study included the measurement of explanatory variables, i.e., psychological dimensions of femininity and masculinity (independent variables), as well as explained variables, i.e., the dimensions of motivation (dependent variables).

## 2. Method

### 2.1. Participants

A total of 49 women aged between 19 and 32 years (M = 22.82; Me = 21; SD = 2.721) representing sports such as football (N = 22), handball (N = 9), hockey (N = 7), volleyball (N = 6), and basketball (N = 5) were studied. The mean training length of the women participants was 9.27 years (Me = 10; SD = 4.310). The group included participants (30%) and medalists (12% of all surveyed women) of championship class competitions (national and European championships).

The second group consisted of 56 men aged 18 to 31 years (M = 21.54; Me = 21; SD = 2.351) training in football (N = 33), hockey (N = 11), volleyball (N = 7), basketball (N = 3), and handball (N = 2). The athletes surveyed were characterized by a training length of 9.84 years on average (Me = 9.5; SD = 4.475). The group included European championship medalists and world championship participants (17% of all surveyed men).

In our research, the criterion for including players from the study was having a minimum of 5 years of training experience, representing their club in national competitions, and participating in training and sports competitions in the senior age category.

The survey was conducted by traditional means, in the presence of the author of the paper. Before completing the questionnaires, subjects were informed of the voluntary nature of their participation in the study, of its anonymity, and that the data obtained would be used exclusively for scientific purposes. The subjects first filled out the Inventory to Assess Psychological Gender (IPP) by Kuczyńska [50], followed by the Sport Motivation Scale (SMS-28) [51,52]. The surveyed players filled in questionnaires before training, which took place at least 2 days after league matches (competition) were held. Such a procedure was aimed at minimizing fatigue, an exhausting (physically and mentally) match unit.

### 2.2. Measurement Tools

The choice of research tools was dictated by the assumption of a relationship between the dimensions of femininity and masculinity and the level of motivation. Therefore, the following research tools were used:

1. Inventory to Assess Psychological Gender (IPP), developed on the basis of the Bem Sex Role Inventory (BSRI) for assessing psychological characteristics [50]. The respondents’ task is to determine the extent to which they are characterized by each of 35 traits, 15 of which reflect the cultural stereotype of femininity and 15 of which reflect the cultural stereotype of masculinity. Respondents refer to the adjectives contained in the questionnaire using a five-point scale. The final score consists of the sum of the points obtained for the items included in the key: femininity scale (K) and masculinity scale (M).

The analysis of the reliability of the questionnaire based on the internal estimation method using the Kuder–Richardson formula showed that the questionnaire is characterized by sufficient reliability for the scales: masculinity (r_tt_ = 0.7834) and femininity (r_tt_ = 0.7856) [50].

2. Sport Motivation Scale (SMS-28) [51], Polish adaptation by Walczak and Tomczak [52], at the core of the tool is the Self-determination theory (STD). The scale contains 28 statements identifying the source of motivation to undertake a given sport. Differentiation in the motivation process is distinguished on the basis of three types of motivation: intrinsic motivation (motivation to know, motivation to experience stimulation, and motivation to accomplish), extrinsic motivation (introjection, identification, and external regulation), and amotivation.

The correlations between the SMS subscales of test and retest were satisfactory, with r = 0.73 for amotivation and r = 0.83 for experience stimulation Moreover, the reliability of each subscale was as follows (Cronbach’s alpha scores): motivation to know—0.81, motivation to accomplish—0.80, motivation to experience stimulation—0.83, identification—0.73, introjection—0.73, external regulation—0.75, and amotivation—0.77 [52].

### 2.3. Statistical Analysis

Statistical analyses were conducted using Statistica 13.3. The Pearson’s r correlation coefficient was used to assess the strength and direction of the relationship between the psychological dimensions of femininity and masculinity and the different forms of motivation and their specific parts. Then, in order to extract the set of best predictors, the stepwise method of progressive selection [53] was applied. Here, a summary regression was performed for the dependent variables, i.e., the selected forms involving the motivation process.

## 3. Results

Firstly, the relationships between the dimensions of psychological femininity and masculinity in the study group of women and the different forms of motivation were analyzed. The results are presented in Table 1.

In the statistical analysis, it was determined that, in women athletes, the dimension of psychological masculinity was significantly and positively related to the motive to know (r = 0.472; *p <* 0.001), motivation to accomplish (r = 0.345; *p <* 0.05), and motivation to experience stimulation (r = 0.420; *p <* 0.01), as well as the overall dimension of intrinsic motivation (r = 0.446; *p <* 0.001) and the overall level of extrinsic motivation (r = 0.334; *p <* 0.05).

Analogous relationships were then analyzed in the group of male subjects training team sports games. The results are presented in Table 2.

The study indicated that, in the group of athletes, the level of psychological femininity was negatively related to amotivation (r = −0.350; *p <* 0.01), while psychological masculinity entered into positive and significant relationships with the motivation to know (r = 0.343; *p <* 0.01), motivation to accomplish (r = 0.407; *p <* 0.01), motivation to experience stimulation (r = 0.367; *p <* 0.01), the overall dimension of extrinsic motivation (r = 0.427; *p <* 0.001), the introjection motive (r = 0.339; *p <* 0.05), and the overall level of extrinsic motivation (r = 0.290; *p <* 0.05).

The analyses presented next are intended to indicate what proportion of the variation in motivation could be explained by variation in the dimensions of femininity and masculinity studied. The data in Table 3, Table 4, Table 5, Table 6 and Table 7 show the coefficients of determination determining the percentage of variance for the selected variables explained—intrinsic motivation, extrinsic motivation, and amotivation.

The characteristics indicated in Table 3 together explained 20.5% of the variance (sample rate) of the presented level of extrinsic motivation (adjusted coefficient indicates 17%) (F(2.46) = 5.929; *p <* 0.001). The masculinity introduced in the first step explained about 20% of the variance. The change in the coefficient of determination after adding another predictor no longer achieved significance at the *p <* 0.05 level.

In the final model obtained (one variable), there was only a positive contribution of psychological masculinity (Beta = 0.46; *p <* 0.01) to the presented level of intrinsic motivation in the studied group of women.

The two indicated subject variables explained a total of 15.4% of the variance (sample rate) (adjusted coefficient indicates 11.7%) (F(2.46) = 4.180; *p <* 0.05) of the level of extrinsic motivation presented by women coaching team sports games (Table 4), whereas masculinity, introduced in the first step, explained approximately 11% of the variance. The change in the coefficient of determination after the addition of another predictor no longer achieved significance at the *p <* 0.05 level.

In the final model obtained (one variable), there was only a positive contribution of psychological masculinity (Beta = 0.37; *p <* 0.01) to the presented level of extrinsic motivation in the women respondents.

The characteristics indicated in Table 5 together explained 20% of the variance (sample rate) of the presented level of intrinsic motivation (adjusted coefficient indicates 17%) (F(2.53) = 6.613; *p <* 0.01). The masculinity introduced in the first step explained about 18% of the variance. The change in the coefficient of determination after adding another predictor no longer achieved significance at the *p <* 0.05 level.

In the final model obtained (one variable), there was only a positive contribution of psychological masculinity (Beta = 0.41; *p <* 0.01) to the presented level of intrinsic motivation in the studied group of men.

The indicated characteristics together explained 9.1% of the variance (sample rate) of the presented level of extrinsic motivation (adjusted coefficient indicates 5.7%) (F(2.53) = 6.659; *p <* 0.001), whereas the masculinity introduced in the first step explained approximately 8% of the variance (Table 6). The change in the coefficient of determination after adding another predictor no longer achieved significance at the *p <* 0.05 level.

In the final model obtained (one variable), there was only a positive contribution of psychological masculinity (Beta = 0.28; *p <* 0.05) to the presented level of extrinsic motivation in the studied group of men.

The characteristics indicated in Table 7 explained 15.6% of the variance (sample rate) of the presented level of amotivation (adjusted coefficient indicates 12.7%) (F(2.53) = 4.879; *p <* 0.05). The femininity introduced in the first step explained about 12% of the variance, and the change in the coefficient of determination after adding another predictor no longer achieved significance at the *p <* 0.05 level.

In the final model obtained (one variable), there was only a positive contribution of psychological masculinity (Beta = −0.33; *p <* 0.05) to the presented level of extrinsic motivation in the male group studied.

## 4. Discussion

Human gender is a category differentiated by each society [54], and patterns of femininity and masculinity are a historically shaped dichotomy [55]. This may be indicated by the multitude of gender-defining categories created [56] and stereotypes related to the sociocultural roles performed by women and men [57,58]. Some of these descriptions include the different types of task involvement [21,22,49] of individuals characterized by different levels of femininity or masculinity; hence, the main objective of the study carried out was to identify the relationships of the two specified psychological dimensions with different forms of motivation.

First of all—on the basis of our own research—it could be determined that the psychological dimension of femininity was (in the male group) positively related to the dimension of amotivation, i.e., the lack of perception of a relationship between one’s action and the outcome. The rationale for these results can be based on the characteristics of the psychological dimension of femininity, i.e., emotionality, shifting priorities to interpersonal relationships, strong expressions, or non-abusive behavior caused by aggression [18,20]; these aspects of functioning may hinder effective task performance in situations of direct sport competition, among others. The manifestation of a predisposition related to psychological femininity may also reduce the male athlete’s ability to perceive his agency and affect his sense of self-efficacy by perceiving various aspects of his activity in an emotional way, including such responses to challenges or threats that arise. The level of a trait such as femininity may, therefore, have a negative impact on the success of an athlete’s actions, especially in competition fought with other men in the area of team sports, who may tend to pursue goals through the use of aggressive behavior [29,30]. Moreover, as shown by other analyses, among men training in team sports games, the greatest number of people was characterized by a specific psychological gender, i.e., exhibiting behaviors typical of the definition of masculinity [59].

Studies have also shown that the psychological dimension of masculinity—in both women and male athletes—positively influences the motivation to know (curiosity, exploration, and desire to learn), motivation to accomplish (mastery, efficiency, and task orientation), motivation to experience stimulation (experiencing exciting sensations), and the general level of intrinsic motivation, which is related to the feeling of satisfaction and/or the pursuit of personal development [60], as well as reflects key needs that participants often see as key to their sporting motivation [61]. Similar to the above discussion, the present result can be substantiated by the description of a profile—this time—of psychological masculinity, which is related to the pursuit of leadership, competition, self-confidence, resourcefulness, or task orientation [12,20], as also shown in relation to stress coping styles by Bojkowski, Walczak, and Tomczak [62]. Individuals possessing a high intensity of this psychological dimension, as well as socially and culturally related attributes, may, through self-activity, seek to expand individual capabilities, and these may result from and at the same time be a catalyst for further progress in learning, pursuing mastery, or experiencing a sense of excitement about pursuing specific goals and being able to achieve them efficiently in team sports. Such a conclusion may support the view on the legitimacy of taking care of the effectiveness of training processes, including mixed selection, based on the assessment of technical, tactical, and psychological needs and predispositions of players to given sports activities [63].

In a final step, it was also determined that the psychological dimension of masculinity—in both men and women—is related to the general dimension of extrinsic motivation (undertaking an activity based on external factors), including in male athletes, also influencing the level of introjection motive, i.e., the process of integrating accepted patterns and rules into the subject’s personality [25]. The result of this analysis can be described through the dimension of the traditional view of masculinity, from the aspects of self-perception and functioning such as self-confidence or competitiveness [64]. Justification for the analysis can also be found in the view of Gråstén [65], who linked extrinsic motivation directly to masculine traits and a tendency toward social comparisons, as well as researchers who identified that those who train team sports games tend to be characterized by greater ego-oriented attitudes [66]. These, in turn, foster public demonstration of one’s skills in front of others and comparison of one’s competence against theirs [66]. Thus, it is determined that masculinity as a dimension that influences action, as well as the individual’s self-perception, can influence the tendency to externalize one’s individual aptitude, to compare oneself, to strive for competition, and, in extreme situations, to ostentatiously dominate one’s superiority over a sporting opponent, which is not so much an expression of self-confidence but of arrogance (a negative trait). Such a view is in line with scientific reports that substantiate that men and, thus, individuals typically characterized by higher levels of psychological masculinity often cite as a motive for participating in physical activity the opportunity to participate in direct competition and to be victorious [65,67], as well as the demonstration of their own strength, endurance, courage, and fighting skills [68,69], i.e., those traits that appear to be associated with an emphasis on masculinity status. The introjection dimension may, in turn, be influenced by the individual’s experience of a desire to partially internalize the rules and norms in force in a given environment or sports team, which may result in increased integration of the task group in which a particular athlete functions. The key issue here, however, may be to thoroughly understand the mechanisms underlying the motivation to engage in team sports in different periods of life or at different levels of sport advancement [61].

## 5. Conclusions

The study determined the existence of relationships between culturally and socially shaped dimensions of femininity and masculinity and the types of motivation and their specific dimensions. The final conclusions, therefore, concern the possible selection or verification of players representing team sports games in terms of psychological dimensions of femininity and masculinity, drawing attention to their, as described in the study, influence on the possibility of satisfying innate developmental requirements (described in the self-determination theory) and basic psychological needs, based on the assimilation of applicable social rules [23,24,52].

## 6. Practical Implications

The conducted research has shown that psychological masculinity is significantly related to the general level of external motivation and, especially worth emphasizing, internal motivation (the most expected form of motivation), including its components (motivation to know, motivation to accomplish, and motivation to experience stimulation). For this reason, the process of improvement in sport should support the development of those characteristics of athletes of both sexes, which are based on the skills of competition, leadership, self-confidence, and task orientation [20], which can help to develop satisfaction with undertaken activities and foster a sense of agency.

## Figures and Tables

**Table 1:** Relationships between the levels of psychological femininity and masculinity and dimensions of motivation in the group of women (women athletes) training in team sports games studied—Pearson’s r correlation coefficients.

Variable	Correlations N = 49	
	F	M
KN	r = −0.048; *p =* 0.742	**r = 0.472; *p =* 0.001**
ACC	r = −0.058; *p =* 0.692	**r = 0.345; *p =* 0.015**
ES	r = 0.102; *p =* 0.049	**r = 0.420; *p =* 0.003**
INTER	r = 0.002; *p =* 0.987	**r = 0.446; *p =* 0.001**
ID	r = −0.158; *p =* 0.279	r = 0.258; *p =* 0.074
IN	r = −0.067; *p =* 0.649	r = 0.260; *p =* 0.071
RZ	r = −0.113; *p =* 0.439	r = 0.252; *p =* 0.080
EXTER	r = −0.144; *p =* 0.324	**r = 0.334; *p =* 0.019**
AMOT	r = −0.185; *p =* 0.203	r = −0.090; *p =* 0.538

Bold values denote statistical significance at the *p* < 0.05 level. F—femininity; M—masculinity; KN—motivation to know; ACC—motivation to accomplish; ES—motivation to experience stimulation; INTER—intrinsic motivation; ID—identification; IN—introjection; RZ—external regulation; EXTER—external motivation; AMOT—amotivation.

**Table 2:** Relationships between the levels of psychological femininity and masculinity and dimensions of motivation in a group of male respondents (players) training team sortie games—Pearson r correlation coefficients.

Variable	Correlations N = 56	
	F	M
KN	r = 0.066; *p =* 0.631	**r = 0.344; *p =* 0.009**
ACC	r = 0.169; *p =* 0.214	**r = 0.407; *p =* 0.002**
ES	r = 0.248; *p =* 0.066	**r = 0.367; *p =* 0.005**
INTER	r = 0.182; *p =* 0.179	**r = 0.427; *p =* 0.001**
ID	r = 0.197; *p =* 0.146	r = 0.249; *p =* 0.065
IN	r = 0.108; *p =* 0.429	**r = 0.339; *p =* 0.011**
RZ	r = −0.005; *p =* 0.971	r = 0.130; *p =* 0.340
EXTER	r = 0.117; *p =* 0.389	**r = 0.290; *p =* 0.030**
AMOT	**r = −0.350; *p =* 0.008**	r = −0.223; *p =* 0.098

Bold values denote statistical significance at the *p* < 0.05 level. F—femininity; M—masculinity; KN—motivation to know; ACC—motivation to accomplish; ES—motivation to experience stimulation; INTER—intrinsic motivation; ID—identification; IN—introjection; RZ—external regulation; EXTER—external motivation; AMOT—amotivation.

**Table 3:** Multiple regression explaining variation in intrinsic motivation based on psychological femininity and masculinity in the group of women respondents.

N = 49	Intrinsic Motivation R = 0.453; R^2^ = 0.205; Adjusted R^2^ = 0.170 F(2.46) = 5.929; *p <* 0.001; SE of Estimate: 11.057					
	b*	SE of b*	b	SE of b	t(46)	*p*
Intercept			20.64373	16.44008	1.255695	0.215567
M	**0.459945**	**0.133566**	**0.85656**	**0.24874**	**3.443588**	**0.001234**
F	−0.078890	0.133566	−0.12529	0.21212	−0.590649	0.557646

Bold values denote statistical significance at the *p* < 0.05 level. M—masculinity; F—femininity.

**Table 4:** Multiple regression explaining the variation in the presented level of extrinsic motivation based on psychological femininity and masculinity in the group of women studied.

N = 49	External Motivation R = 0.392; R^2^ = 0.154; Adjusted R^2^ = 0.117 F(2.46) = 4.180; *p <* 0.05; SE of Estimate: 9.979					
	b*	SE of b*	b	SE of b	t(46)	*p*
Intercept			37.02729	14.83714	2.49558	0.016224
M	**0.370576**	**0.137798**	**0.60371**	**0.22449**	**2.68927**	**0.009942**
F	−0.209418	0.137798	−0.29094	0.19144	−1.51975	0.135418

Bold values denote statistical significance at the *p* < 0.05 level. M—masculinity; F—femininity.

**Table 5:** Multiple regression explaining variation in intrinsic motivation based on psychological femininity and masculinity in the group of male respondents.

N = 56	Intrinsic Motivation R = 0.447; R^2^ = 0.200; Adjusted R^2^ = 0.170 F(2.53) = 6.613; *p <* 0.01; SE of Estimate: 9.744					
	b*	SE of b*	b	SE of b	t(53)	*p*
Intercept			12.03200	15.07051	0.798381	0.428213
M	**0.411186**	**0.123796**	**0.71496**	**0.21525**	**3.321476**	**0.001627**
F	0.132122	0.123796	0.22606	0.21182	1.067259	0.290692

Bold values denote statistical significance at the *p* < 0.05 level. M—masculinity; F—femininity.

**Table 6:** Multiple regression explaining variation in extrinsic motivation based on psychological femininity and masculinity in the male study group.

N = 56	External Motivation R = 0.302; R^2^ = 0.091; Adjusted R^2^ = 0.057 F(2.53) = 2.659; *p <* 0.07936; SE of Estimate: 10.703					
	b*	SE of b*	b	SE of b	t(53)	*p*
Intercept			20.92985	16.55406	1.264333	0.211642
M	**0.280303**	**0.131924**	**0.50238**	**0.23644**	**2.124729**	**0.038291**
F	0.083332	0.131924	0.14697	0.23267	0.631668	0.530319

Bold values denote statistical significance at the *p* < 0.05 level. M—masculinity; F—femininity.

**Table 7:** Multiple regression explaining variation in amotivation based on psychological femininity and masculinity in the male study group.

N = 56	Amotivation R = 0.394; R^2^ = 0.156; Adjusted R^2^ = 0.127 F(2.53) = 4.879; *p <* 0.05; SE of Estimate: 5.553					
	b*	SE of b*	b	SE of b	t(53)	*p*
Intercept			35.14407	8.587694	4.09238	0.000146
F	**−0.327438**	**0.127171**	**−0.31078**	**0.120701**	**−2.57479**	**0.012859**
M	−0.183539	0.127171	−0.17703	0.122658	−1.44324	0.154836

Bold values denote statistical significance at the *p* < 0.05 level. F—femininity; M—masculinity.

## Data Availability

The data presented in this study are available on request from the corresponding author.
